# Plasma Biomarker Profiling of 2-Hydroxypropyl-β-Cyclodextrin (HPβCD) Treatment in an Aged Mouse Model of Ischemic Stroke

**DOI:** 10.3390/ijms262210814

**Published:** 2025-11-07

**Authors:** Danielle A. Becktel, Jennifer B. Frye, Elizabeth H. Le, Rick G. Schnellmann, Kristian P. Doyle

**Affiliations:** 1 Department of Immunobiology, College of Medicine, University of Arizona, Tucson, AZ 85724, USA; dbecktel@arizona.edu (D.A.B.); jabeisch@arizona.edu (J.B.F.); elizabethle@arizona.edu (E.H.L.); 2 Department of Pharmacology and Toxicology, R. Ken Coit College of Pharmacy, University of Arizona, Tucson, AZ 85724, USA; schnell@pharmacy.arizona.edu; 3 Coit Center for Longevity and Neurotherapeutics, R. Ken Coit College of Pharmacy, University of Arizona, Tucson, AZ 85724, USA; 4 BIO5 Institute, College of Medicine, University of Arizona, Tucson, AZ 85724, USA; 5 Department of Neurology, College of Medicine, University of Arizona, Tucson, AZ 85724, USA; 6 Arizona Center on Aging, University of Arizona, Tucson, AZ 85724, USA; 7 Department of Psychology, College of Science, University of Arizona, Tucson, AZ 85724, USA; 8 Department of Neurosurgery, College of Medicine, University of Arizona, Tucson, AZ 85724, USA

**Keywords:** stroke, 2-hydroxypropyl-β-cyclodextrin, cyclodextrin, biomarkers, lipids, oxylipins, oxysterols, cholesterol

## Abstract

Lipid debris generated after ischemic stroke overwhelms myeloid cells, leading to foam cell-like dysfunction and chronic neuroinflammation. 2-hydroxypropyl-β-cyclodextrin (HPβCD), a cholesterol-mobilizing agent, has been shown to improve recovery and reduce chronic inflammation after stroke by enhancing lipid processing and cholesterol efflux in infarcts. To identify plasma biomarkers of HPβCD activity and gain mechanistic insight into lipid pathway modulation, aged (21-month-old) male mice underwent the distal middle cerebral artery occlusion + hypoxia (DH) model of stroke and received 2 g/kg HPβCD twice daily beginning 1 d after stroke. Plasma metabolomic and lipidomic profiling was performed 4 d after stroke using untargeted (Global Discovery) and targeted (Complex Lipid, Oxysterols, and Lipid Mediators of Inflammation) panels. Acute neuroprotection was assessed by magnetic resonance imaging (MRI) quantification of infarct, ventricle, and hippocampus volumes 2 d after stroke and by plasma neurofilament light (NfL) levels 4 d after stroke. HPβCD treatment did not provide acute neuroprotection; however, HPβCD did induce distinct plasma metabolomic and lipidomic signatures, including decreases in sphingolipids, cholesterol, long-chain fatty acids, 4β-hydroxycholesterol, 7-dehydrocholesterol, and 8-dehydrocholesterol and increases in 27-hydroxycholesterol and 7α-hydroxy-3-oxo-4-cholestenoic acid (7-HOCA), consistent with enhanced cholesterol efflux and metabolism. Pro-inflammatory oxylipins were also suppressed by HPβCD treatment. These results support the role of HPβCD in promoting lipid debris clearance and suppressing inflammatory lipid pathways after stroke and, together with prior studies demonstrating improved long-term recovery, highlight HPβCD as a biomarker-supported therapeutic candidate for stroke recovery.

## 1. Introduction

Ischemic stroke initiates a multifaceted cascade of secondary injury processes that extends beyond the primary vascular occlusion [1,2]. A key downstream event is the substantial release of lipid debris from degenerating neurons, myelin, and other central nervous system (CNS) cells [3,4]. Cholesterol, sphingolipids, and oxidized fatty acids liberated from the infarcted tissue accumulate within infiltrating monocytes and resident microglia, promoting a “foamy” or lipid-laden phenotype [5]. We and others have shown that this lipid overload disrupts myeloid lipid catabolism, thereby impairing phagocytosis, delaying resolution of inflammation, and sustaining neurotoxic signaling within the infarcted brain [6,7,8,9,10].

In prior work, we also identified a distinct plasma metabolomic and lipidomic signature associated with this process in aged mice, characterized by elevations in oxylipins and myelin-derived sphingolipids [11]. This signature indicates that dysregulated lipid metabolism in the brain is reflected systemically through alterations in circulating metabolites and lipids. These observations further suggest that therapies aimed at improving lipid mobilization and breakdown may provide a promising strategy for post-stroke treatment, offering both a potential intervention and circulating biomarkers to monitor treatment efficacy [8,12].

One such candidate is 2-hydroxypropyl-β-cyclodextrin (HPβCD), a cyclic oligosaccharide with high cholesterol-binding affinity that has demonstrated efficacy in models of Niemann-Pick disease type C (NPC) and atherosclerosis [13,14,15,16]. In previous studies, we found that HPβCD improves long-term functional recovery and reduces chronic inflammation in young adult and aged mouse models of stroke [7]. Transcriptomic analyses of stroke infarcts from HPβCD-treated mice revealed upregulation of lipid processing and cholesterol efflux pathways, suggesting that HPβCD alleviates lipid overload within myeloid cells [7]. However, the mechanisms underlying these beneficial effects remain incompletely understood, and no circulating biomarkers currently exist to monitor HPβCD activity.

The present study aimed to (i) determine whether HPβCD confers acute neuroprotection after stroke, (ii) identify plasma metabolites and lipids that could serve as biomarkers of HPβCD activity and provide translational indicators of target engagement and treatment efficacy, and (iii) elucidate its mechanism of action by defining the systemic metabolic and inflammatory pathways influenced by HPβCD.

To address these aims, aged (21-month-old) male mice were subjected to distal middle cerebral artery occlusion + hypoxia (DH) stroke and randomized to receive either vehicle or HPβCD (2 g/kg, s.c.) twice daily beginning 1 d after stroke. *T*_2_-weighted magnetic resonance imaging (MRI) was conducted 2 d after stroke to evaluate acute neuroprotective effects. Plasma was collected 4 d after stroke for downstream analyses of neurofilament light (NfL), metabolomic, and lipidomic profiles. This integrative approach enabled identification of circulating biomarkers of HPβCD activity, characterization of its impact on lipid catabolic and inflammatory pathways, and assessment of potential neuroprotective effects in the acute phase after stroke.

## 2. Results

### 2.1. HPβCD Treatment Does Not Confer Acute Neuroprotection

**Figure 1:**
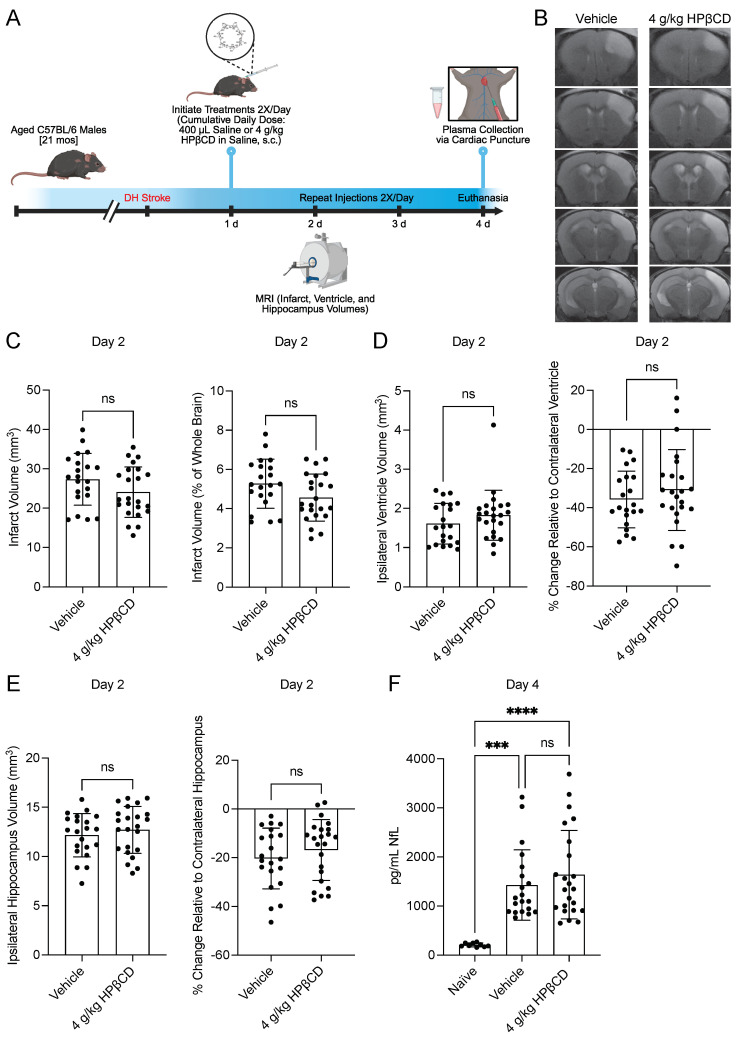
2-hydroxypropyl-β-cyclodextrin (HPβCD) treatment does not confer acute neuroprotection. (**A**) Experimental design, 21-month-old male mice were subjected to distal middle cerebral artery occlusion + hypoxia (DH) stroke and randomized to receive either vehicle or HPβCD (2 g/kg, s.c.) twice daily beginning 1 d after stroke. *T*_2_-weighted magnetic resonance imaging (MRI) was performed 2 d after stroke to assess acute pathological sequelae. Plasma was collected at euthanasia, performed 30 min after administration of the final dose, 4 d after stroke. (**B**) Representative *T*_2_-weighted MR images acquired 2 d after stroke show similar infarct, hippocampus, and ventricle volumes in vehicle-injected and HPβCD-treated mice. (**C**) HPβCD treatment did not significantly affect infarct volumes 2 d after stroke (*n* = 21–23; unpaired *t* tests; *p* > 0.05). (**D**) HPβCD treatment did not significantly alter ipsilateral ventricle volumes 2 d after stroke; however, ipsilateral ventricular compression consistent with mass effect from cortical edema was observed (*n* = 21–23; Mann–Whitney test, panel 1; unpaired *t* test, panel 2; *p* > 0.05). (**E**) HPβCD treatment did not significantly alter ipsilateral hippocampus volumes 2 d after stroke; however, ipsilateral hippocampal compression consistent with mass effect from cortical edema was observed (*n* = 21–23; unpaired *t* tests; *p* > 0.05). (**F**) Plasma neurofilament light (NfL) levels were markedly elevated in mice 4 d after stroke compared with naïve controls, but NfL levels did not differ significantly between vehicle-injected and HPβCD-treated mice (*n* = 9–23; Kruskal–Wallis test, **** *p* < 0.0001; Dunn’s multiple comparisons test; *** *p* < 0.001, **** *p* < 0.0001). Data are presented as mean ± SD. ns, not significant.

To identify plasma biomarkers of HPβCD activity and evaluate its effects on acute ischemic injury, aged (21-month-old) male mice were subjected to DH stroke and randomized to receive either vehicle or HPβCD (2 g/kg, s.c.) twice daily beginning 1 d after stroke. *T*_2_-weighted MRI was performed 2 d after stroke to quantify infarct, hippocampus, and ventricle volumes. Plasma was collected 4 d after stroke, 30 min after the final vehicle or HPβCD dose was administered, for downstream analyses of neurofilament light (NfL), metabolomic, and lipidomic profiles (Figure 1A).

*T*_2_-weighted MRI was performed 2 d after stroke to assess the impact of three doses of HPβCD treatment on acute pathological sequelae. The quantification of *T*_2_-weighted MRI scans revealed that HPβCD treatment did not significantly affect infarct volumes 2 d after stroke (Figure 1B,C). Similarly, HPβCD treatment did not significantly alter ipsilateral ventricle or hippocampus volumes; however, ipsilateral ventricular and hippocampal compression consistent with mass effect from cortical edema was observed (Figure 1D,E). These results indicate that HPβCD does not alter gross infarct size or MRI-derived structural outcomes in the acute phase after stroke.

To further assess neuronal injury, plasma NfL concentrations were measured 4 d after stroke. Plasma NfL levels were markedly elevated in mice 4 d after stroke compared with naïve controls, confirming axonal damage in the DH stroke model. However, NfL levels did not differ significantly between vehicle-injected and HPβCD-treated mice (Figure 1F). Together, these results demonstrate that while stroke induces robust neuronal injury, HPβCD treatment does not confer acute neuroprotection.

### 2.2. HPβCD Treatment Induces a Distinct Plasma Metabolomic Signature 4 d After Stroke

**Figure 2:**
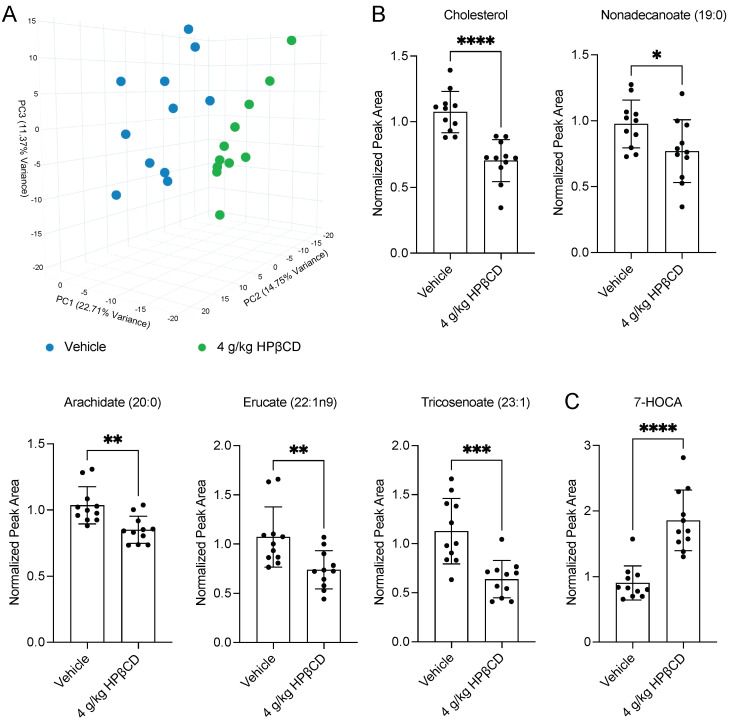
HPβCD treatment induces a distinct plasma metabolomic signature 4 d after stroke. (**A**) Unsupervised principal component analysis (PCA) of the global untargeted metabolomic dataset revealed clear separation between vehicle-injected and HPβCD-treated mice, indicating that HPβCD treatment induces a distinct shift in circulating plasma metabolites 4 d after stroke. (**B**) HPβCD treatment significantly decreased plasma levels of cholesterol, long-chain saturated fatty acids, including nonadecanoate (19:0) and arachidate (20:0), and long-chain monounsaturated fatty acids, including erucate (22:1n9) and tricosenoate (23:1) (cholesterol, nonadecanoate (19:0), tricosenoate (23:1): *n* = 11; unpaired *t* tests; * *p* < 0.05, *** *p* < 0.001, **** *p* < 0.0001; arachidate (20:0), erucate (22:1n9): *n* = 11; Mann–Whitney tests; ** *p* < 0.01). (**C**) In contrast, HPβCD treatment significantly increased plasma levels of 7α-hydroxy-3-oxo-4-cholestenoic acid (7-HOCA), an exportable intermediate in the oxidative clearance of brain cholesterol (*n* = 11; unpaired *t* test; **** *p* < 0.0001). Data are presented as mean ± SD.

To determine whether HPβCD treatment induces a distinct plasma metabolomic signature in the acute phase after stroke, plasma samples were collected at euthanasia 4 d after stroke. These samples were sent to Metabolon, Inc. for global untargeted metabolomic analysis using the Global Discovery Panel. Unsupervised principal component analysis (PCA) of the resultant dataset revealed clear separation between vehicle-injected and HPβCD-treated mice 4 d after stroke (Figure 2A). This distinct clustering indicates that HPβCD treatment induces a reproducible shift in the circulating metabolite profile, establishing a global framework for identifying specific plasma biomarkers of HPβCD activity.

Further assessment of circulating metabolites revealed that HPβCD treatment reduced several lipid species derived from the breakdown of myelin and other cellular membranes, which contribute to lipid overload and myeloid cell dysfunction after stroke [8]. Specifically, HPβCD treatment significantly decreased plasma levels of cholesterol, long-chain saturated fatty acids, including nonadecanoate (19:0) and arachidate (20:0), and long-chain monounsaturated fatty acids, including erucate (22:1n9) and tricosenoate (23:1) (Figure 2B). In contrast, HPβCD treatment significantly increased plasma levels of 7α-hydroxy-3-oxo-4-cholestenoic acid (7-HOCA), an exportable intermediate in the oxidative clearance of brain cholesterol [17] (Figure 2C). Elevated plasma 7-HOCA suggests that HPβCD facilitates the oxidative conversion and peripheral export of cholesterol derived from lipid debris after stroke, thereby promoting systemic clearance and limiting its accumulation in myeloid cells. However, further studies are necessary to confirm this proposed mechanism.

### 2.3. HPβCD Treatment Decreases Plasma Sphingolipid Levels 4 d After Stroke

**Figure 3:**
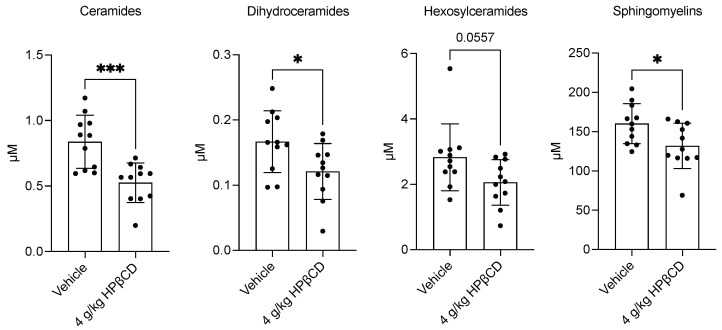
HPβCD treatment decreases plasma sphingolipid levels 4 d after stroke. HPβCD treatment decreased plasma levels of multiple sphingolipid classes, including ceramides, dihydroceramides, hexosylceramides, and sphingomyelins (ceramides, dihydroceramides, sphingomyelins: *n* = 11; unpaired *t* tests; * *p* < 0.05, *** *p* < 0.001; hexosylceramides: *n* = 11; Mann–Whitney test; *p* = 0.0557). Data are presented as mean ± SD.

To further characterize lipidomic alterations associated with HPβCD treatment in the acute phase after stroke, plasma samples were submitted to Metabolon, Inc. for targeted lipidomic analysis using the Complex Lipid Panel. In parallel with reductions in cholesterol, long-chain saturated fatty acids, and long-chain monounsaturated fatty acids, HPβCD treatment also decreased plasma levels of multiple sphingolipid classes, including ceramides, dihydroceramides, hexosylceramides, and sphingomyelins (Figure 3). Sphingolipids and cholesterol, principal constituents of myelin membranes, were reduced in plasma, supporting the hypothesis that HPβCD facilitates mobilization and processing of lipid debris generated after stroke. The resulting metabolomic and lipidomic signature, marked by decreased sphingolipids and cholesterol alongside increased 7-HOCA, provides circulating biomarkers of HPβCD activity and suggests enhanced turnover and clearance of myelin- and membrane-derived lipids in response to treatment. This mechanism warrants further experimental validation.

### 2.4. HPβCD Treatment Alters Cholesterol Metabolism Through Oxysterol Pathways 4 d After Stroke

**Figure 4:**
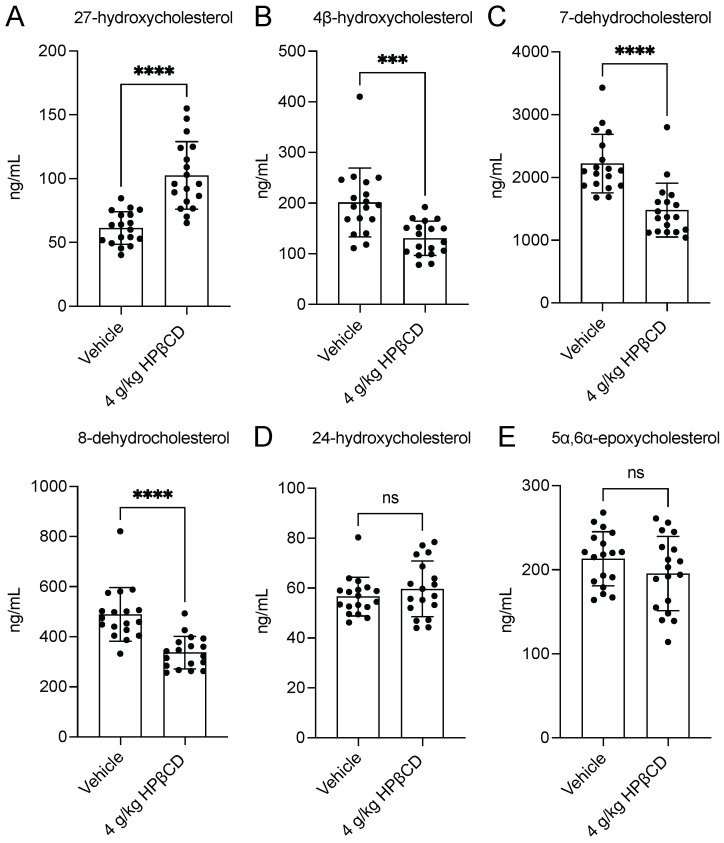
HPβCD treatment alters cholesterol metabolism through oxysterol pathways 4 d after stroke. (**A**) HPβCD treatment significantly increased plasma levels of 27-hydroxycholesterol, consistent with enhanced cholesterol efflux and cytochrome P450 (CYP)27A1-dependent catabolism (*n* = 18; unpaired *t* test; **** *p* < 0.0001). (**B**) In parallel, plasma levels of 4β-hydroxycholesterol, a sensitive indicator of hepatic CYP3A enzyme induction, were decreased by HPβCD treatment (*n* = 18; Mann–Whitney test; *** *p* < 0.001). (**C**) Plasma levels of 7-dehydrocholesterol and 8-dehydrocholesterol, terminal intermediates in the cholesterol biosynthetic pathway, were also decreased by HPβCD treatment (*n* = 18; Mann–Whitney tests; **** *p* < 0.0001). (**D**,**E**) In contrast, plasma 24-hydroxycholesterol, produced by CYP46A1 in both neuronal and peripheral tissues in mice, and 5α,6α-epoxycholesterol, formed primarily by nonenzymatic autoxidation of cholesterol, were unchanged by HPβCD treatment (*n* = 18; unpaired *t* tests; *p* > 0.05). Data are presented as mean ± SD. ns, not significant.

Together, these coordinated shifts in plasma cholesterol and sphingolipids prompted a detailed analysis of oxysterol pathways to determine whether HPβCD redirects debris-derived cholesterol toward specific routes of catabolism and efflux. Plasma samples were submitted to Metabolon, Inc. for assessment using the Targeted Oxysterol Panel, which revealed that HPβCD altered multiple branches of cholesterol metabolism. HPβCD treatment significantly increased plasma 27-hydroxycholesterol, consistent with enhanced cholesterol efflux and cytochrome P450 (CYP)27A1-dependent catabolism [18] (Figure 4A). In parallel, plasma 4β-hydroxycholesterol, a sensitive indicator of hepatic CYP3A enzyme induction, was decreased [19] (Figure 4B). The reciprocal changes in 27-hydroxycholesterol and 4β-hydroxycholesterol indicate a shift from CYP3A-mediated hydroxylation to mitochondrial CYP27A1-dependent catabolism. Additionally, plasma levels of 7-dehydrocholesterol and 8-dehydrocholesterol, terminal intermediates in the cholesterol biosynthetic pathway, were also decreased, consistent with reduced accumulation of late-stage synthesis intermediates [20,21] (Figure 4C). We interpret these alterations as potential evidence of feedback suppression of cholesterol biosynthesis secondary to increased cholesterol mobilization and enhanced catabolic flux through CYP27A1-dependent pathways. However, further studies are needed to experimentally validate these proposed mechanisms.

In contrast, plasma 24-hydroxycholesterol, produced by CYP46A1 in both neuronal and peripheral tissues in mice, remained unchanged by HPβCD treatment [22,23,24] (Figure 4D). Plasma levels of 5α,6α-epoxycholesterol, formed primarily by nonenzymatic autoxidation of cholesterol, were also unaffected, indicating that mobilization of cholesterol by HPβCD did not increase nonenzymatic oxidation of circulating sterols [25] (Figure 4E). Instead, the observed increases in 27-hydroxycholesterol and 7-HOCA, coupled with decreases in cholesterol biosynthetic precursors, indicate selective engagement of enzymatic side-chain oxidation and efflux pathways. Collectively, the plasma oxysterol profile suggests that HPβCD promotes cholesterol clearance through oxidative catabolism and reduces de novo cholesterol synthesis, without evidence of increased nonenzymatic oxidation or substantial disruption of neuronal cholesterol-derived metabolites.

### 2.5. HPβCD Treatment Attenuates Pro-Inflammatory Plasma Oxylipin Production 4 d After Stroke

**Figure 5:**
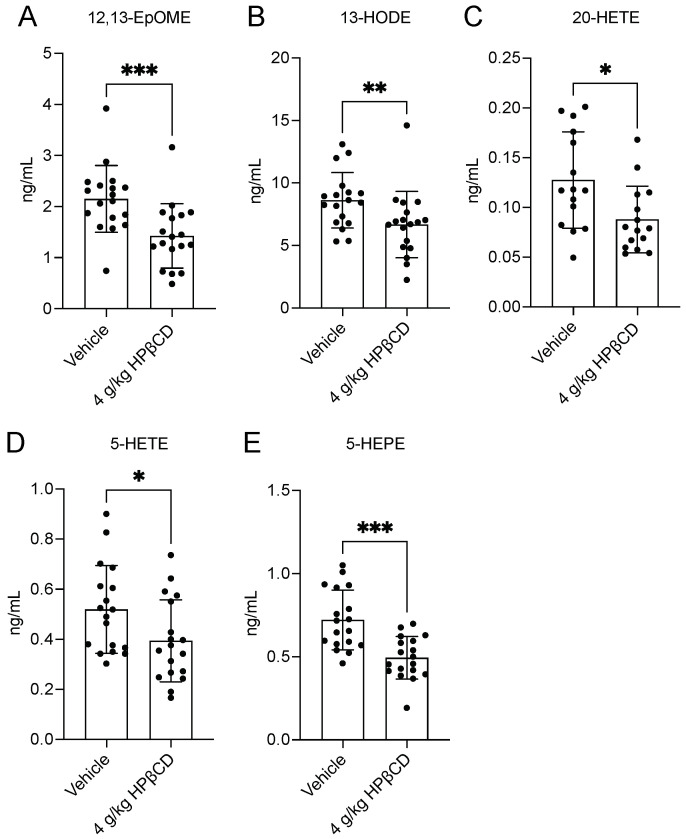
HPβCD treatment attenuates pro-inflammatory plasma oxylipin production 4 d after stroke. (**A**,**B**) Plasma levels of linoleic acid-derived oxylipins, 12,13-epoxyoctadecenoic acid (12,13-EpOME; isoleukotoxin) and 13-hydroxyoctadecadienoic acid (13-HODE), were markedly decreased by HPβCD treatment (*n* = 18; Mann–Whitney tests; ** *p* < 0.01, *** *p* < 0.001). (**C**) Plasma 20-hydroxyeicosatetraenoic acid (20-HETE), an arachidonic acid metabolite generated by CYP4A/4F activity, was decreased by HPβCD treatment (*n* = 15; unpaired *t* test; * *p* < 0.05). (**D**,**E**) HPβCD treatment also significantly reduced plasma levels of 5-lipoxygenase (LOX)-derived products, including 5-hydroxyeicosatetraenoic acid (5-HETE) and 5-hydroxyeicosapentaenoic acid (5-HEPE) (5-HETE: *n* = 18; Mann–Whitney test; * *p* < 0.05; 5-HEPE: *n* = 18; unpaired *t* test; *** *p* < 0.001). Data are presented as mean ± SD.

Building on the observed alterations in cholesterol metabolism, plasma oxylipin pathways were analyzed, as oxidized fatty acid mediators represent key downstream effectors of lipid-driven inflammation after stroke. Plasma samples were submitted to Metabolon, Inc. for assessment using the Lipid Mediators of Inflammation Panel, which demonstrated that HPβCD significantly reduced multiple pro-inflammatory oxylipins. Plasma levels of linoleic acid-derived metabolites, including 12,13-epoxyoctadecenoic acid (12,13-EpOME; isoleukotoxin) and 13-hydroxyoctadecadienoic acid (13-HODE), were markedly decreased by HPβCD treatment (Figure 5A,B). Plasma 20-hydroxyeicosatetraenoic acid (20-HETE), an arachidonic acid metabolite generated by CYP4A/4F activity, was also decreased, along with 5-lipoxygenase (LOX)-derived products, including 5-hydroxyeicosatetraenoic acid (5-HETE) and 5-hydroxyeicosapentaenoic acid (5-HEPE) [26] (Figure 5C–E). Together, these reductions indicate that HPβCD attenuates pro-inflammatory plasma oxylipin production, potentially by limiting substrate availability or modulating enzyme activity.

## 3. Discussion

### 3.1. Summary

The present study demonstrates that HPβCD, administered beginning 1 d after experimental stroke, produces a unique plasma metabolomic signature, identifies biomarkers of activity, and offers insights into its mechanisms of action. Although early administration of HPβCD did not confer acute neuroprotection, these findings build on our previous studies demonstrating that lipid debris overwhelms myeloid cells, leading to foam cell-like dysfunction and chronic neuroinflammation [6,7,8]. By mobilizing and processing this debris, HPβCD appears to restore lipid homeostasis, dampen oxylipin production, and enhance cholesterol metabolism after stroke.

### 3.2. Plasma Biomarkers of HPβCD Activity

A central objective of the present study was to identify plasma metabolites and lipids that could serve as biomarkers of HPβCD activity. Plasma metabolomic and lipidomic profiling revealed consistent reductions in sphingolipids, including ceramides, dihydroceramides, hexosylceramides, and sphingomyelins, alongside decreased cholesterol and increased 7-HOCA. Plasma 7-HOCA, a product of cholesterol side-chain oxidation, reflects flux into bile acid-directed catabolism and serves as a pharmacodynamic marker of cholesterol mobilization and turnover [17,27]. In addition, plasma oxysterol profiling demonstrated increased 27-hydroxycholesterol and decreased 4β-hydroxycholesterol, 7-dehydrocholesterol, and 8-dehydrocholesterol, while 24-hydroxycholesterol remained stable. These observations indicate that HPβCD enhances cholesterol efflux and enzymatic oxidation of cholesterol to soluble metabolites while limiting accumulation of biosynthetic intermediates and inflammatory catabolites [18,19,20,21]. Collectively, these plasma biomarkers represent translationally relevant indicators of HPβCD activity that could be applied in clinical studies to confirm target engagement and optimize dosing strategies.

### 3.3. Insights into HPβCD Mechanisms of Action

Beyond biomarker discovery, these data demonstrate that HPβCD influences post-stroke pathophysiology through effects on oxylipin production. Oxylipins, potent lipid mediators generated by the oxidation of polyunsaturated fatty acids, are rapidly produced from the lipid debris released after stroke and have critical roles in vascular and inflammatory responses [28,29]. HPβCD treatment reduced plasma levels of several pro-inflammatory oxylipins, including 12,13-EpOME, 13-HODE, 20-HETE, and 5-LOX products such as 5-HETE and 5-HEPE, suggesting suppression of pathways linked to vascular dysfunction, leukocyte recruitment, and tissue injury [30,31]. Arachidonic acid-derived mediators, including 20-HETE and 5-HETE, promote endothelial dysfunction, vasoconstriction, and leukocyte infiltration, whereas linoleic acid-derived mediators, including 13-HODE and 12,13-EpOME, amplify myeloid cell activation and cytokine release [30,32,33,34]. HPβCD may indirectly suppress these oxylipin pathways, possibly by limiting enzymatic lipid oxidation, thereby attenuating neurovascular stress and dampening inflammatory amplification loops.

**Figure 6:**
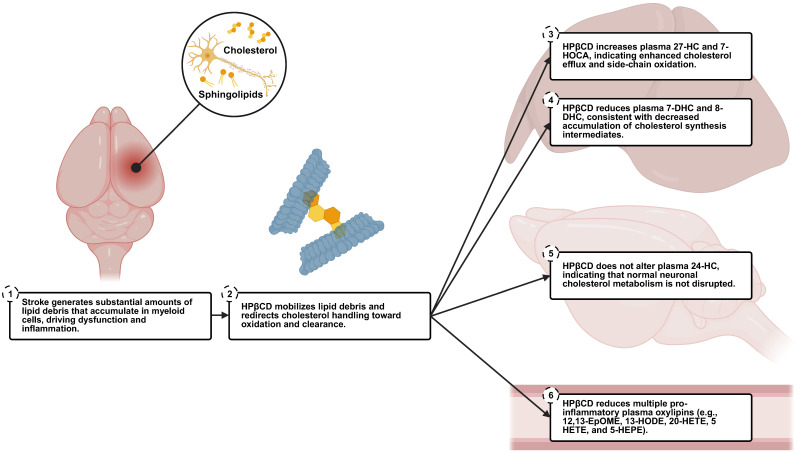
Proposed HPβCD mechanisms of action after stroke. Stroke generates substantial amounts of lipid debris that accumulate in myeloid cells, driving dysfunction and inflammation. HPβCD mobilizes this lipid debris and redirects cholesterol handling toward oxidation and clearance through several mechanisms. Treatment with HPβCD increases plasma 27-hydroxycholesterol (27-HC) and 7α-hydroxy-3-oxo-4-cholestenoic acid (7-HOCA), indicating enhanced cholesterol efflux and side-chain oxidation, while reducing plasma 7-dehydrocholesterol (7-DHC) and 8-dehydrocholesterol (8-DHC), consistent with decreased accumulation of cholesterol synthesis intermediates. In contrast, HPβCD does not alter plasma 24-hydroxycholesterol (24-HC), predominantly produced by neurons via CYP46A1 and representing the main route of cholesterol turnover across the blood–brain barrier (BBB), indicating that HPβCD selectively targets pathological lipid pools without interfering with normal neuronal cholesterol metabolism. HPβCD also reduces multiple pro-inflammatory plasma oxylipins, including 12,13-epoxyoctadecenoic acid (12,13-EpOME), 13-hydroxyoctadecadienoic acid (13-HODE), 20-hydroxyeicosatetraenoic acid (20-HETE), 5-hydroxyeicosatetraenoic acid (5-HETE), and 5-hydroxyeicosapentaenoic acid (5-HEPE). Collectively, these effects support a model in which HPβCD alleviates lipid overload in immune cells, reprograms cholesterol handling toward clearance in the liver, normalizes lipid mediators of inflammation, and fosters a less injurious inflammatory milieu.

In addition, plasma oxysterol profiling suggests that HPβCD promotes the oxidative metabolism and efflux of cholesterol liberated from lipid debris after stroke, while suppressing de novo cholesterol synthesis. Increases in plasma 27-hydroxycholesterol and 7-HOCA, alongside decreases in 7-dehydrocholesterol and 8-dehydrocholesterol, indicate engagement of the acidic bile acid pathway and systemic feedback on cholesterol biosynthesis, plausibly mediated through liver regulatory mechanisms such as liver X receptor (LXR) signaling and sterol regulatory element-binding protein 2 (SREBP2) suppression [20,21,27,35,36,37]. The stable levels of plasma 24-hydroxycholesterol, predominantly produced by neurons via CYP46A1 and representing the main route of cholesterol turnover across the blood–brain barrier (BBB), indicate that HPβCD selectively targets pathological lipid pools without interfering with normal neuronal cholesterol metabolism [23,24,38]. Collectively, these observations support a model in which HPβCD alleviates lipid overload in immune cells, reprograms cholesterol handling toward clearance in the liver, normalizes lipid mediators of inflammation, and fosters a less injurious inflammatory milieu (Figure 6).

### 3.4. Lack of Acute Neuroprotective Effects of HPβCD

Despite pronounced effects on systemic lipid metabolism and oxylipin production, acute measures of ischemic injury, including infarct, hippocampus, and ventricle volumes 2 d after stroke and plasma NfL levels 4 d after stroke, remained unaltered by HPβCD treatment. These results suggest that HPβCD does not confer protection against infarct expansion or axonal injury in the DH stroke model but instead exerts therapeutic effects through promoting lipid debris clearance and suppressing inflammatory pathways related to lipid metabolism. This interpretation aligns with our previous studies demonstrating that HPβCD improves long-term outcomes by targeting lipid-laden myeloid cells and attenuating inflammation [7]. Therefore, HPβCD may act primarily by shaping the subacute and chronic injury environments rather than by altering the immediate trajectory of infarct growth or acute neuronal loss.

### 3.5. Translational Significance

The translational implications of these results are notable. Oxysterols such as 27-hydroxycholesterol and 7-HOCA, which are measurable in human plasma, could serve as pharmacodynamic readouts of HPβCD activity in clinical studies [17,22]. When combined with reductions in sphingolipids and pro-inflammatory oxylipins, these biomarkers may yield panels that capture both mechanistic and efficacy signals of HPβCD treatment. Integrating these molecular readouts with advanced imaging techniques, such as translocator protein (TSPO) positron emission tomography (PET) and dynamic contrast-enhanced (DCE) MRI to assess neuroinflammation and BBB integrity, could provide an even more comprehensive evaluation of treatment response and therapeutic impact.

Although HPβCD did not reduce acute infarct size or plasma NfL, its modulation of lipid metabolism and inflammatory mediators positions it as a therapy that shapes the subacute and chronic injury environments, highlighting its potential for biomarker-guided translation to human stroke studies. Importantly, HPβCD is approved by the U.S. Food and Drug Administration (FDA) as an inert excipient and has undergone extensive safety and toxicity studies; however, optimal dosing strategies for its use as an active pharmaceutical ingredient in ischemic stroke remain to be established [39].

### 3.6. Limitations and Future Directions

Several methodological limitations should be considered when interpreting these findings. First, plasma metabolomic and lipidomic analyses provide broad systemic insight but cannot identify the specific tissues or cell types responsible for the observed alterations. Direct evaluation of lipid handling in myeloid cells, endothelial cells, and hepatocytes, combined with complementary metabolomic and lipidomic profiling of brain and liver tissues, will be needed to confirm the proposed mechanisms.

Second, these experiments were performed exclusively in male mice. As sex-dependent differences in cholesterol metabolism and endolysosomal function could influence HPβCD efficacy, parallel studies in female mice are essential to determine whether similar therapeutic effects occur [40].

Third, although oxysterols such as 24-hydroxycholesterol and 27-hydroxycholesterol are established circulating indicators of sterol metabolism in humans, clinical translation of the broader biomarker panel may be constrained by current assay availability and sensitivity [18,22]. Further validation in human stroke cohorts will also be necessary to determine whether the observed biomarker alterations correspond to clinically meaningful improvements in recovery.

Fourth, all plasma samples were collected at a single, standardized time point (30 min after the final dose), which may have limited detection of more transient or delayed biomarker responses. In addition, methodological variability across the four analytical platforms, including differences in sample preparation, instrument configuration, and lipid extraction procedures, complicates direct cross-panel comparisons.

Despite these limitations, the metabolomic signature identified here may have clinical utility for personalizing post-stroke rehabilitation. If validated in patients, this circulating biomarker profile could help identify individuals most likely to benefit from HPβCD or related lipid-modulating therapies, monitor biochemical responses during treatment, and guide adjunctive interventions that target post-stroke inflammation.

## 4. Materials and Methods

### 4.1. Animals

The present study was designed in a manner consistent with the ARRIVE and RIGOR guidelines [41,42,43]. Sample sizes were determined by power analysis based on plasma cholesterol as the primary outcome measure (α = 0.05, β = 0.05, and power = 0.95), which indicated a minimum requirement of 11 mice per experimental group. In total, 48 aged (21-month-old) wild-type male C57BL/6 mice were procured from the National Institute on Aging. Mice were randomly allocated to cages and housed in a temperature- and humidity-controlled facility under a 12 h light/dark cycle with ad libitum access to food and water. All experimental procedures were conducted in accordance with the National Institutes of Health Guide for the Care and Use of Laboratory Animals and were approved by the University of Arizona Institutional Animal Care and Use Committee (Protocol Code: 13-487; Date Approved: 8 June 2022). Mice were excluded if ischemia could not be confirmed by MRI (*n* = 2) or if post-operative mortality occurred (*n* = 2). At the conclusion of each experiment (30 min after administration of the final dose), mice were anesthetized by isoflurane inhalation and positioned supine. The thoracic surface was disinfected with 70% ethanol, and a midline incision was made to expose the heart. Using a sterile syringe fitted with a fine-gauge needle, blood was collected from the left ventricle by gentle aspiration. After exsanguination, mice were perfused transcardially with 0.9% saline. Blood was transferred immediately to EDTA-coated microcentrifuge tubes on ice and then centrifuged at 4 °C for 10 min at 5000× *g* to obtain plasma. Plasma aliquots were distributed to each of the four panels conducted by Metabolon, Inc. (Morrisville, NC, USA) and stored at −80 °C until analysis.

### 4.2. Stroke Surgeries

Stroke was induced in mice using the distal middle cerebral artery (MCA) occlusion + hypoxia (DH) model. The DH model yields a reproducible cortical infarct encompassing approximately 24% of the ipsilateral hemisphere, primarily within the somatosensory cortex, while maintaining low variability and high survival rates. The inclusion of hypoxia is necessary, as C57BL/6 mice subjected to distal MCA occlusion alone develop only small cortical infarcts. Detailed descriptions and validation of this model have been published previously [44,45]. To induce stroke, mice were anesthetized by isoflurane inhalation and maintained at 37 ± 1 °C using a feedback-controlled heating system. Pre-operative analgesia consisted of a single subcutaneous (s.c.) injection of buprenorphine hydrochloride (0.1 mg/kg) dissolved in 0.9% sterile saline, USP. After preparing the surgical site, a skin incision was made over the right temporoparietal region between the orbit and the ear to expose the skull. Under an operating microscope, a small cranial opening was created with a high-speed microdrill over the visually identified distal branch of the MCA, near the parietal cerebral artery. Permanent occlusion of the MCA was performed by electrocauterization with a fine-tip cauterizer. Surgical wounds were sealed with Surgi-lock 2oc tissue adhesive. Immediately after surgery, mice were placed in a temperature-controlled hypoxia chamber containing 11% O_2_ and 89% N_2_ for 45 min. Post-operative analgesia was provided with an extended-release formulation of buprenorphine (Ethiqa XR; Fidelis Pharmaceuticals, North Brunswick, NJ, USA; 3.25 mg/kg, s.c.) administered 1 d after surgery.

### 4.3. Drug Treatments

Mice were randomized to receive either vehicle or 2-hydroxypropyl-β-cyclodextrin (HPβCD) (2 g/kg, s.c.) twice daily beginning 1 d after stroke. Treatments were repeated each morning and afternoon for four consecutive days (i.e., eight total doses). Treatment frequency and duration were informed by our previous observation that plasma levels of myelin- and membrane-derived lipids rise acutely after stroke but return to baseline levels within one week [11]. Vehicle-injected mice received 0.9% sterile saline, USP (400 µL/day cumulative), whereas HPβCD-treated mice received an equivalent volume of HPβCD solution (4 g/kg/day cumulative) prepared in 0.9% sterile saline, USP, at a final concentration of 350 mg/mL. HPβCD powder (KLEPTOSE^®^ HPB Parenteral Grade; Roquette, Lestrem, France; Product Code: 346111) was used. MRI was performed after administration of the third dose, and euthanasia was conducted 30 min after administration of the eighth and final dose.

### 4.4. MRI

MRI was performed 2 d after stroke to quantify infarct, ventricle, hippocampus, and whole brain volumes. Imaging was conducted on a Bruker BioSpec 70/20 7.0T scanner (Billerica, MA, USA) with ParaVision-360.3.2 software and a 4-channel phased-array mouse coil. Mice were positioned in a cradle fitted with a stereotaxic frame and feedback-controlled heating system that maintained body temperature at 37 ± 1 °C. Respiratory rate was continuously monitored using a pressure probe, and anesthesia was maintained by isoflurane inhalation throughout image acquisition. High-resolution structural images were obtained using a *T*_2_-weighted RARE Bruker pulse sequence with the following parameters: repetition time (*TR*) = 2500 ms; flip angle = 30°; RARE factor = 8; matrix size = 256 × 256; averages = 2; field of view = 20 mm × 20 mm; slice thickness = 0.8 mm; number of slices = 15; acquisition time = 2 min 40 s. Each region of interest was manually delineated on *T*_2_-weighted MR images using Mango v4.1. Image processing and volumetric analyses were conducted by an investigator blinded to experimental groups and independent of all treatment administrations.

### 4.5. Plasma Neurofilament Light Analysis (Quanterix^®^)

Neurofilament light (NfL) was quantified in mouse plasma samples using the Simoa^®^ (Single Molecule Array) NF-Light v2 Advantage Assay (Quanterix^®^, Billerica, MA, USA; Cat. No. 104073) according to manufacturer instructions. Measurements for all calibrator, control, and experimental samples were performed in duplicate on the Simoa^®^ SR-X Instrument (Quanterix^®^, Billerica, MA, USA). This assay employs a two-step digital immunoassay format based on paramagnetic capture beads coated with anti-NfL antibodies. During the first incubation, NfL present in the sample binds to these capture beads along with a biotin-labeled detection antibody, forming a sandwich complex. After unbound components are washed away, streptavidin–β-galactosidase (SBG) is added to associate with the biotinylated antibody, thereby enzymatically tagging each captured complex. The beads are then washed again and resuspended in a resorufin β-D-galactopyranoside (RGP) substrate solution before being loaded into the microwell array of the Simoa^®^ disc. Each microwell isolates an individual bead, allowing fluorescent signal generation when β-galactosidase cleaves the RGP substrate. The Simoa^®^ optical detection system then counts fluorescent wells, enabling quantification of NfL at single-molecule sensitivity.

### 4.6. Plasma Global Untargeted Metabolomic Analysis—Global Discovery Panel (Metabolon, Inc.)

A total of 870 metabolites (i.e., 735 named/identified and 135 unnamed) were quantified in mouse plasma samples using ultra-performance liquid chromatography coupled to tandem mass spectrometry (UPLC-MS/MS). To remove proteins, dissociate small molecules bound to or trapped within the precipitated protein matrix, and recover chemically diverse metabolites, proteins were precipitated with methanol under vigorous shaking for 2 min, followed by centrifugation. The resulting extract was divided into multiple fractions: two for analysis by separate reverse phase (RP)/UPLC-MS/MS methods with positive ion mode electrospray ionization (ESI), one for analysis by RP/UPLC-MS/MS with negative ion mode ESI, one for hydrophilic interaction liquid chromatography (HILIC)/UPLC-MS/MS with negative ion mode ESI, while the remaining fractions were reserved for backup. All methods used a Waters ACQUITY UPLC (Milford, MA, USA) and a Thermo Scientific Q-Exactive high resolution/accurate mass spectrometer (Waltham, MA, USA) interfaced with a heated electrospray ionization (HESI-II) source and Orbitrap mass analyzer operated at 35,000 mass resolution [46]. The dried sample extracts were then reconstituted in solvents suitable for each of the four methods. Each reconstitution solvent contained a set of standards at fixed concentrations to ensure injection and chromatographic consistency. One aliquot was analyzed using acidic positive ion conditions, optimized for more hydrophilic compounds (PosEarly). In this method, the extract was gradient-eluted from a C18 column (Waters UPLC BEH C18-2.1x100 mm, 1.7 µm) with water and methanol, containing 0.05% perfluoropentanoic acid (PFPA) and 0.1% formic acid (FA). Another aliquot was also analyzed under acidic positive ion conditions but was chromatographically optimized for more hydrophobic compounds (PosLate). In this method, the extract was gradient eluted from the same aforementioned C18 column using methanol, acetonitrile, water, 0.05% PFPA, and 0.01% FA, operating at an overall higher organic content. Another aliquot was analyzed under basic negative ion optimized conditions using a separate dedicated C18 column (Neg). The basic extracts were gradient eluted from the column with methanol and water, but with 6.5 mM ammonium bicarbonate at pH 8. The fourth aliquot was analyzed via negative ionization after elution from a HILIC column (Waters UPLC BEH Amide 2.1x150 mm, 1.7 µm) using a gradient of water and acetonitrile with 10 mM ammonium formate at pH 10.8 (HILIC). The MS analysis alternated between MS and data-dependent MS^n^ scans with dynamic exclusion. Metabolites were identified by comparing them to library entries of purified standards or recurring unknown entities. Metabolon, Inc. maintains a library based on authenticated standards that includes retention time/index (RI), mass-to-charge ratio (*m*/*z*), and fragmentation data for all molecules in the library. Furthermore, biochemical identifications relied on three criteria: RI within a narrow range of the proposed identification, accurate mass matching the library within +/−10 ppm, and MS/MS forward and reverse scores indicating similarity between the experimental data and authentic standards. Peaks were quantified by calculating the area-under-the-curve and normalized to account for day-to-day variation. All analyses were performed by investigators blinded to experimental groups.

### 4.7. Plasma Targeted Lipidomic Analysis—Complex Lipid Panel (Metabolon, Inc.)

875 named/identified lipids were quantified in mouse plasma samples using differential mobility spectroscopy. In brief, lipids were extracted from the samples using a modified version of the method described by Matyash et al., which involves methyl-*tert*-butyl ether and deuterated internal standards [47]. Extracts were concentrated under nitrogen and reconstituted in 0.25 mL of 10 mM ammonium acetate dichloromethane:methanol (50:50). Extracts were then transferred to inserts and placed in vials for infusion-MS analysis, performed on a Shimadzu LC with nano PEEK tubing (Kyoto, Japan) and SCIEX SelexION 5500 QTRAP (Framingham, MA, USA). Samples were analyzed using both positive and negative mode electrospray. Individual lipid species were quantified by multiplying the peak area ratios of target compounds and their assigned internal standards by the internal standard concentration in the sample. Each lipid class concentration was calculated by summing all lipids belonging to that class. All analyses were performed by investigators blinded to experimental groups.

### 4.8. Plasma Targeted Lipidomic Analysis—Oxysterols Targeted Panel (Metabolon, Inc.)

12 oxysterols and related sterols of biological significance were quantified in mouse plasma samples using LC-MS/MS. For the analysis of 24-hydroxycholesterol, 27-hydroxycholesterol, 4β-hydroxycholesterol, 5α,6α-epoxycholesterol, 7-dehydrocholesterol, and 8-dehydrocholesterol, 50 μL of sample was spiked with internal standard solution, hydrolyzed, extracted by liquid–liquid extraction with hexanes, and then derivatized. After derivatization, a second liquid–liquid extraction with hexanes was carried out. After centrifugation, the hexanes layer was evaporated to dryness, reconstituted in acidified methanol, and injected into an Agilent 1290 (Santa Clara, CA, USA)/SCIEX 5500 QTRAP LC-MS/MS system with a Waters ACQUITY BEH Shield RP18 Column. The mass spectrometer operated in positive mode with ESI. Peak areas of the analyte product ions were measured against those of the corresponding internal standard product ions. Quantitation used a weighted linear least squares analysis from fortified calibration standards prepared alongside the experimental samples. LC-MS/MS raw data were collected with SCIEX Analyst 1.7.3 software and processed using SCIEX OS-MQ v3.1.6. Data reduction was conducted using Microsoft Excel for Microsoft 365 MSO. All analyses were performed by investigators blinded to experimental groups.

### 4.9. Plasma Targeted Lipidomic Analysis—Lipid Mediators of Inflammation Targeted Panel (Metabolon, Inc.)

A total of 58 lipid mediators of inflammation were quantified in mouse plasma samples using LC-MS/MS. Briefly, plasma was spiked with stable labelled internal standards, extracted, and subjected to protein precipitation with an organic solvent. After centrifugation, an aliquot of the supernatant was injected into an Agilent 1290 Infinity II/AB SCIEX QTRAP 7500 LC-MS/MS system equipped with a Waters CORTECS T3 column. For the analysis of 12,13-EpOME, 13-HODE, 20-HETE, 5-HETE, and 5-HEPE, the mass spectrometer was operated in negative mode using ESI. The peak area of the individual analyte product ions was measured relative to the peak area of the product ions of pre-determined internal standards. Quantitation was performed using a weighted linear or quadratic least squares analysis generated from fortified calibration standards prepared before each run. LC-MS/MS raw data were collected and processed with SCIEX OS-MQ software v3.0. Data reduction was conducted using Microsoft Excel for Office 365 MSO. All analyses were performed by investigators blinded to experimental groups.

### 4.10. Statistical Analysis

Statistical analyses were performed using GraphPad Prism v10.5.0. Data distributions were assessed for normality using the Shapiro–Wilk (W) test. When datasets satisfied the assumption of normality, parametric tests were applied, specifically a two-tailed, unpaired *t* test for comparisons between two groups or an ordinary one-way ANOVA followed by Dunnett’s multiple comparisons test for comparisons between multiple groups. For datasets that did not meet the normality assumption, nonparametric tests were applied, specifically a Mann–Whitney test for comparisons between two groups or a Kruskal–Wallis test followed by Dunn’s multiple comparisons test for comparisons between multiple groups. Statistical significance was defined as *p* < 0.05. Data are presented as mean ± standard deviation (SD). Effect sizes and confidence intervals are reported in Supplementary Materials Table S9.

## 5. Conclusions

In conclusion, HPβCD treatment after stroke remodels the plasma lipidome, reduces markers of lipid-laden myeloid cells, and reprograms cholesterol metabolism via oxysterol pathways. HPβCD treatment also suppresses pro-inflammatory oxylipins that drive vascular dysfunction and leukocyte recruitment. Although acute infarct size and plasma NfL levels were unchanged, these observations underscore the potential of HPβCD to facilitate lipid debris clearance, reduce chronic myeloid cell dysfunction, and dampen sustained inflammation after stroke. The resulting biomarker signatures provide insight into these mechanisms of action and offer translationally relevant readouts of activity, supporting further development of HPβCD as a strategy to attenuate chronic inflammation and improve recovery after stroke.

## Data Availability

The original contributions presented in this study are included in the article and Supplementary Materials Tables S1–S9. Further inquiries can be directed to the corresponding author.

## Supplementary Materials

The following supporting information can be downloaded at: https://www.mdpi.com/article/10.3390/ijms262210814/s1.

## Abbreviations

The following abbreviations are used in this manuscript:

5-HEPE	5-hydroxyeicosapentaenoic acid
5-HETE	5-hydroxyeicosatetraenoic acid
7-DHC	7-dehydrocholesterol
7-HOCA	7α-hydroxy-3-oxo-4-cholestenoic acid
8-DHC	8-dehydrocholesterol
12,13-EpOME	12,13-epoxyoctadecenoic acid
13-HODE	13-hydroxyoctadecadienoic acid
20-HETE	20-hydroxyeicosatetraenoic acid
24-HC	24-hydroxycholesterol
27-HC	27-hydroxycholesterol
ANOVA	Analysis of variance
BBB	Blood–brain barrier
CNS	Central nervous system
CYP	Cytochrome P450
DH	Distal middle cerebral artery occlusion + hypoxia
EDTA	Ethylenediaminetetraacetic acid
ESI	Electrospray ionization
FA	Formic acid
FDA	Food and Drug Administration
HILIC	Hydrophilic interaction liquid chromatography
HPβCD	2-hydroxypropyl-β-cyclodextrin
LOX	Lipoxygenase
LXR	Liver X receptor
MCA	Middle cerebral artery
MRI	Magnetic resonance imaging
MS	Mass spectrometry
NfL	Neurofilament light
ns	Not significant
NPC	Niemann-Pick disease type C
PCA	Principal component analysis
PET	Positron emission tomography
PFPA	Perfluoropentanoic acid
RGP	Resorufin ß-D-galactopyranoside
RI	Retention time/index
RP	Reverse phase
SBG	Streptavidin-ß-galactosidase
s.c.	Subcutaneous
SD	Standard deviation
SREBP2	Sterol regulatory element-binding protein 2
TSPO	Translocator protein
UPLC-MS/MS	Ultra-performance liquid chromatography-tandem mass spectrometry
